# Alternative transcription increases isoform complexity in Long Non-Coding RNAs and alters their functions in cancer

**DOI:** 10.1016/j.ncrna.2025.04.008

**Published:** 2025-05-03

**Authors:** Max Bone, Gareth J. Inman

**Affiliations:** a School of Cancer Sciences, University of Glasgow, United Kingdom; b Cancer Research UK Scotland Institute, Glasgow, United Kingdom

**Keywords:** Cancer, Non Coding RNA, isoforms, Alternative transcription, Antisense oligonucleotides, lncRNA

## Abstract

Transcriptional start and end variance, a less-explored aspect of lncRNA biology, is a critical determinant of isoform diversity in human RNA. While alternative splicing (AS) has been extensively studied as a mechanism of isoform generation, differences in transcriptional start and termination site usage—whether from distinct promoters or varying initiation events at the same core promoter—contribute more to isoform diversity than alternative splicing. In the context of long non-coding RNAs (lncRNAs), even subtle alterations to transcriptional start and end sites can induce significant changes in the structural and functional capacities of individual lncRNA isoforms.

This review highlights the underappreciated realm of transcriptional start and end variance in lncRNAs, exploring its pivotal role in shaping the diversity of lncRNA transcripts. In cancer, where lncRNAs are increasingly recognised as key players in tumorigenesis, understanding the ramifications of transcriptional start and end variance is crucial. With single nucleotide alterations capable of influencing the folding energy, shape, stability, and function of a lncRNA molecules, significant changes to transcriptional regulation may lead to aberrant isoforms with implications for cancer initiation, progression, and potentially, its treatment.

As lncRNAs emerge as therapeutic targets, particularly with the advancement of antisense oligonucleotide (ASO) technologies, it becomes crucial to understand the regulatory landscape of transcriptional variation among lncRNA isoforms, to ensure selective targeting of oncogenic transcripts while sparing those with normal physiological functions. By highlighting the significance of transcriptional start and end site variation as major contributors to lncRNA diversity, the potential exploitation for precision therapeutic interventions in the field of non-coding RNA cancer research can be expanded.

## 1 Introduction

The generation of nascent RNA from DNA transcription is a complex and non-linear event in human cells. Despite its complex nature, transcription is a highly regulated process that can result in the generation of multiple RNA products from a singular DNA locus, enhancing biological storage efficiency by providing a single gene with the flexibility to generate diverse RNA molecules. This flexibility allows cells to adapt to different conditions without needing more genetic material. It arises from variations in transcriptional processes within pre-RNA, even before post-transcriptional modifications like splicing [[1], [2], [3], [4]] (see Table 1).

**Table 1:** An abbreviated summary of discussed lncRNA, their isoforms mentioned, and oncogenic roles.

Gene locus	Isoform	Oncogenic roles	References
**MALAT1**	Full-length MALAT1	Extensive; including regulation of splicing and metastasis, and interactions with epigenetic regulators.	[[62], [63], [64], [65]]
**MALAT1**	mascRNA	Promotes TRAF6 degradation; enhances IFN signalling and inflammation resolution.	[69,70]
**NEAT1**	NEAT1_1	Supports proliferation and bone metastasis of prostate cancers.	[82]
**NEAT1**	NEAT1_2	Essential for paraspeckle formation; promotes cancer progression via miRNA sponging (miR-106b-5p, and miR-491).	[[73], [74], [75], [76],^84^,^85^]
**GAS5**	GAS5 (exon 12 truncated)	Unidentified isoform specific roles however likely altered GR decoy capacity, potential roles in related cancers.	[[88], [89], [90], [91], [92], [93], [94], [95], [96], [97], [98], [99], [100]]
**GAS5**	GAS5 (full length exon 12)	Downregulated in cancers; involved in apoptosis via GR decoy activity, many alternative cancer roles.	[[88], [89], [90], [91], [92], [93], [94], [95], [96], [97], [98], [99], [100]]
**LINC00941**	LINC00941-201	Unidentified isoform specific roles, *LINC00941 s*tabilizes ANXA2 protein; enhances tumour invasiveness, promotes EMT and suppresses SPRR5. Acts in a variety of cancers.	[[104], [105], [106], [107], [108]]
**LINC00941**	LINC00941-207	Unidentified isoform specific roles, *LINC00941 s*tabilizes ANXA2 protein; enhances tumour invasiveness, promotes EMT and suppresses SPRR5. Acts in a variety of cancers.	[[104], [105], [106], [107], [108]]
**PVT1**	lncPVT1 (linear)	Sponges miR-29c; promotes angiogenesis and tumour progression.	[121,123]
**PVT1**	circPVT1	Sponges miR-125; enhances proliferation; prognostic in gastric cancer.	[122,[125], [126], [127], [128]]
**CCAT1**	CCAT1-L	Promotes MYC expression via chromatin looping; nuclear retention.	[134,[139], [140], [141]]
**CCAT1**	CCAT1-S	Acts as miRNA sponge; promotes invasion and metastasis; cytoplasmic.	[[143], [144], [145]]

DNA transcriptional variance can generate different RNA isoforms via numerous mechanisms. The transcription start site (TSS) of a particular gene locus is the first nucleotide to be converted into an RNA molecule by RNA polymerase II (RNA Pol II) and is varied when alternative transcriptional initiation (ATI) occurs. This process is in part regulated by a promotor region, a short sequence of nucleotides upstream genetically of a specific locus [[5], [6], [7]]. These sequences typically range from 100 to 1000bp of DNA in length and act as a recruitment agent for transcriptional machinery, however multiple promoters and enhancers can also exist and at far greater lengths. Typically, specific transcription factors will recognise promotor regions and facilitate the assembly of the pre-initiation complex (PIC), a protein complex that contains RNA Pol II, the mediator complex, and a TFIID/TATA-binding protein (TBP) that allows for attachment to chromatin and subsequent gene transcription [[8], [9], [10], [11]]. Promoters themselves can maintain a flexible TSS architecture that generates RNA isoforms at differential levels in a highly coordinated tissue and environmental dependent manner. Epigenetic modifications, such as DNA methylation, histone modifications, and cellular stressors such as hypoxia, play a central role in regulating promoter activity [12]. DNA methylation at CpG islands within promoters typically represses gene expression by blocking transcription factor binding or recruiting repressive chromatin remodelers. Conversely histone acetylation tends to promote an open chromatin state, enhancing accessibility and transcriptional activation. These modifications collectively shape the transcriptional landscape without altering the underlying DNA sequence [[13], [14], [15], [16]]. Other epigenetic influences affecting alternative transcription include cellular stressors such as temperature, hypoxia, and pathogenic responses [[17], [18], [19], [20]]. Within diseases where genetic mutations are prevalent such as cancers, otherwise stable promotor regions can become genetically altered and subsequent events such as the deregulation of normal gene expression, and the expression of alternative and potentially pathogenic RNA isoforms may be incurred such as in the case of the *TERT* promotor [8,[21], [22], [23], [24]].

RNA isoform variance can also arise as a product of variance at the end of the transcriptional process by a myriad of means known as alternative transcription termination (ATT). This process is commonly tied to specific adenine sequences in the 3′ end of a gene known as polyadenylation sites (PASs) which act as a marker for RNA Pol II to stop processing a DNA strand. Following the transcription of poly-A signals into RNA, the proteins cleavage and polyadenylation specificity factor (CPSF) and cleavage stimulation factor (CstF) undergo a transition from the carboxyl terminal domain of RNA Pol II to bind specifically to the poly-A signal. This binding event initiates the recruitment of additional protein factors to the site such as *XRN2* in the torpedo mechanism of termination, or orchestrate the cleavage of the transcript by disassociating RNA pol II allosterically [[25], [26], [27], [28], [29]]. Several complex and intricate processes can vary during the latter stages of RNA transcription to influence termination events and subsequent RNA isoforms. RNA molecules, including nascent transcripts, can hybridize with DNA forming three stranded R-loops capable of limiting RNA transcription prior to reaching a specific termination site. The formation of R-loops is closely associated with DNA damage and genomic instability, hallmarks frequently observed in cancer [[30], [31], [32], [33], [34], [35]]. Further processes such as heterochromatin patching results in transcriptional pausing, such as via H3K9 methylation which can lead to further alterations in RNA length and termination [36,37].

Overall differential start and termination sites of transcription are responsible for the highest degree of variance in isoforms in RNA in humans [38]. In the context of mRNA this can result in downstream alterations to the structure of proteins as well as the general translational efficiency of mRNA to protein. For non-coding RNA molecules which do not undergo translation or have short ORF's and persist independently of their encoded peptides, their function is wholly dependent on the secondary structure and nucleotide sequence of the molecule, alterations to any part of the mature RNA molecule can have a profound effect on both the structure and function of the molecule [39,40]. Among the non-coding RNA, those above 500 nt in length fall into the category of lncRNA, which are abundant in mammals and are predicted to represent more than 68 % of the total number of entire human transcripts. With the continual discovery of new lncRNA transcripts the total number of these molecules in humans is not known, currently several sources have identified over 100,000 lncRNA genes and more than 300,000 transcripts [[41], [42], [43], [44], [45]]. With advances in the field of next generation sequencing and research into the splicing and transcription of lncRNA continuing to evolve, it is reasonable to assume that many more lncRNA transcripts will be identified and characterised. An abundance of physiological roles has been attributed to lncRNA in terms of remodelling the epigenetic landscape of a cell, often in a highly tissue dependent manner, and can function both in the nucleus and the cytoplasm [39,46]. In the nucleus lncRNA can enact epigenetic properties such as engaging in direct interactions with chromatin to finely regulate gene expression [47]. In addition to their pivotal roles within the nucleus, lncRNAs also undertake significant regulatory roles within the cytoplasm of eukaryotic cells. In the cytoplasm, lncRNAs participate in various cellular processes including post-transcriptional regulation, mRNA stability modulation, micro RNA (miRNA) sponging, translation control, RNA protein binding, and interactions with cellular organelles [[48], [49], [50]]. Given that even minor sequence variations can significantly alter RNA folding and secondary structure, non-coding RNAs such as lncRNAs are particularly susceptible to structural and functional changes [51,52]. Unlike protein-coding RNAs, whose primary function is defined by translation, lncRNAs often exert their roles through specific structural conformations that mediate interactions with proteins, DNA, or other RNAs. As a result, small changes in their sequence whether through alternative splicing, transcriptional variation, or mutation can markedly influence their stability, localization, and regulatory functions. It is therefore of paramount importance to understand how alternative transcription events, however minor, influence the shape, structure, and function of lncRNA isoforms if we are to ever fully understand these elusive molecules.

Dysregulation of lncRNAs has been linked to a wide range of diseases across diverse tissue types, including organ-specific disorders like heart and kidney disease, as well as neurological conditions such as Alzheimer's disease and other neurodegenerative or nerve-related pathologies [[53], [54], [55], [56]]. In recent years, lncRNAs have emerged as key players in cancer biology, driven by growing evidence of their differential expression across tumour types. This has sparked intense interest in understanding how these non-coding transcripts contribute to the regulation of oncogenic pathways [57,58]. With lncRNA sequences directly altering structures and therefore potential functions, recent findings have shown how specific lncRNA isoforms of the same gene can have distinct functional roles in multiple cancers [59,60]. Accurately characterising these molecules, is paramount to understanding how they may be driving neoplastic diseases, and currently with multiple lncRNA being selected for clinical trials for therapeutic options, understanding how different isoforms may be contributing to normal homeostasis and disease may prove to be a significant milestone in this emerging field of cancer treatment [61]. By understanding alternative transcription and its effects on lncRNA isoform variance, more precise therapeutic solutions will theoretically be achieved for managing cancers where lncRNA treatments are a promising avenue of research.

### 1.1 Transcriptional flexibility generates diverse lncRNA isoforms with distinct functions from a single locus

Alternative transcription can generate distinct isoforms of lncRNAs by utilizing different transcription start and end sites. This process results in lncRNAs with unique sequences and structural features, which can lead to diverse functional outcomes. Some of these isoforms can exhibit oncogenic properties, contributing significantly to cancer development and progression. Other isoforms of the same lncRNA gene locus that arise may however prove essential for normal cellular homeostasis and function.

#### 1.1.1 MALAT1 & mascRNA

One such example is the noncoding nuclear-enriched abundant transcript 2 (*NEAT2*) gene or as it is also commonly known *MALAT1* (metastasis associated lung adenocarcinoma transcript 1), which represents a locus that encodes over 66 recognised lncRNA transcript variants with numerous implications in cancer [[62], [63], [64], [65]]. Although one of the first identified lncRNA much about MALAT1 in its roles in normal tissue homeostasis and furthermore its eclectic roles in regulating cancer remain to be fully understood. All identified MALAT1 isoforms lack a 3′ polyadenylation sequence and therefore would expectedly be transcribed inefficiently and subject to 3′–5′ exonucleases mediated degradation. Many MALAT1 transcripts however are subject to a form of noncanonical 3′ end processing where a cytoplasmic tRNA-like region known as *MALAT1*-associated small cytoplasmic RNA (mascRNA) can form stabilising the nascent RNA which protects it from exonuclease mediated degradation and subsequently cleavage via RNase P [[66], [67], [68]]. This means of transcriptional termination in some MALAT1 isoforms is impactful in a specific manner in human tissues. As mascRNA is dissociated from the parent MALAT1 RNA, it can enact oncogenic properties of its own such as its recognised roles as a promotor of proliferation and metastasis in hepatocellular carcinoma via activating the ERK/MAPK signalling pathway, where it was implicated to interact with p-ERK post-transcriptionally [69]. Interestingly, mascRNA also plays a critical functional role by promoting K48-linked ubiquitination and proteasomal degradation of TRAF6. This process suppresses TLR-mediated MyD88-dependent proinflammatory signalling while enhancing TRIF-dependent interferon responses [70]. While not all MALAT1 isoforms encode *mascRNA*, the emergence of two oncogenic molecules from a single lncRNA gene during a single activity of DNA transcription raises intriguing questions about the mechanisms underlying the generation of specific isoforms, especially in the context of cancer. Although the 3′ end of this gene is widely correlated with cancer progression both in terms of MALAT1 and mascRNA expression, a recent study demonstrated that artificially alternatively transcribed 3′ regions of *MALAT1* can prevent retinal oxidative stress from arising in murine models [71]. With new tissue specific and essential functions being ascribed to regions of *MALAT1* that also harbour oncogenic potential, the understanding and selective targeting of specific lncRNA isoforms may prove an important challenge in the recent advances which aim at targeting MALAT1 therapeutically [72].

#### 1.1.2 NEAT1_1 & NEAT1_2

The nuclear paraspeckle assembly transcript 1 (*NEAT1*) is another lncRNA with a diverse abundance of roles in cancer. The overexpression of this gene has been identified within numerous tumour types and has been associated with a poor disease prognosis in oesophageal squamous cell, non-small cell lung, and bladder cancers, as well as many others [[73], [74], [75], [76]]. It's specific overexpression within various cancers is so pronounced, NEAT1 has garnered implications that it’s expression may be used as a form of biomarker for identifying malignant tissues, as was outlined in a recent study in prostate cancer [77]. Alongside extensive implications in cancer, NEAT1 has also been identified as an essential mediator of other cellular processes, a study in 2019 by Zhang et al. uncovered it has essential roles within inflammasome stimulation and identified NEAT1 as a modulator of innate immunity [78]. As NEAT1 is strongly implicated in the progression of various cancers, its underlying molecular mechanisms have been extensively investigated. Notably, this gene gives rise to two structurally and functionally distinct isoforms, NEAT1_1 and NEAT1_2, which originate from alternative 3′ termination events of a shared primary transcript. While the shorter isoform NEAT1_1 (∼3.7 kb) is produced via canonical polyadenylation, NEAT1_2 (∼22.7 kb) results from a unique non-polyadenylated processing pathway involving the formation of a triple helical RNA structure that stabilizes the long transcript [68,79]. This post-transcriptional divergence enables the two isoforms to exert markedly different roles in the cell, particularly in the context of tumorigenesis. Since an investigation in 2009 by Sunwoo et al. [80], NEAT1_2 has widely been established as an essential component of nuclear paraspeckle formation, whilst NEAT1_1 has been identified as diffusing within the nuclei of cells. In the context of cancer, the differences in these two transcripts are apparent [81]. In prostate cancers for example NEAT1_1 has been found to act separately of NEAT1_2 to drive bone metastasis, specifically the m6A modification of NEAT1_1 enhances its oncogenic function by promoting the formation of a CYCLINL1/CDK19 complex, which facilitates RNA Polymerase II Ser2 phosphorylation [82]. The long paraspeckle linked isoform NEAT1_2 has its own distinct identified roles in driving cancers. In HER2 positive breast cancers, NEAT1_2 was associated with high grade disease whereas NEAT1_1 was not [83]. Furthermore, in papillary thyroid cancers, NEAT1_2 has been found to have specific molecular functional roles including the sponging of micro-RNAs, miR-106b-5p, and miR-491 respectively to advance tumour progression to metastatic disease [84,85]. Overall, NEAT1 is a lncRNA of considerable interest across multiple cancer types, adjacent to this are its unique and context-dependent functions, including critical roles in innate immune regulation and paraspeckle formation. Given that these diverse activities are mediated by distinct isoforms generated through alternative transcriptional termination, a deeper understanding of each transcript variant is essential for elucidating the full functional repertoire of NEAT1 in both normal physiology and tumorigenesis.

### 1.2 Transcription variation dictates structure and stability of oncogenic lncRNAs

Transcriptional variation plays a critical role in shaping the structure and stability of oncogenic lncRNAs. Isoforms arising from alternative transcription start or termination sites may exhibit only subtle differences in sequence, yet these can lead to significant changes in RNA folding. Such variations influence thermodynamic properties like folding entropy and free energy, which determine the structural stability and conformational diversity of the RNA. These structural differences are not merely incidental; they directly impact lncRNA function by modulating interactions with proteins, nucleic acids, or chromatin. Even single-nucleotide changes can alter secondary structures enough to shift a lncRNA from a regulatory to an oncogenic role, highlighting the importance of understanding isoform-specific structural dynamics in cancer biology [86,87].

#### 1.2.1 GAS5

**Fig. 1:**
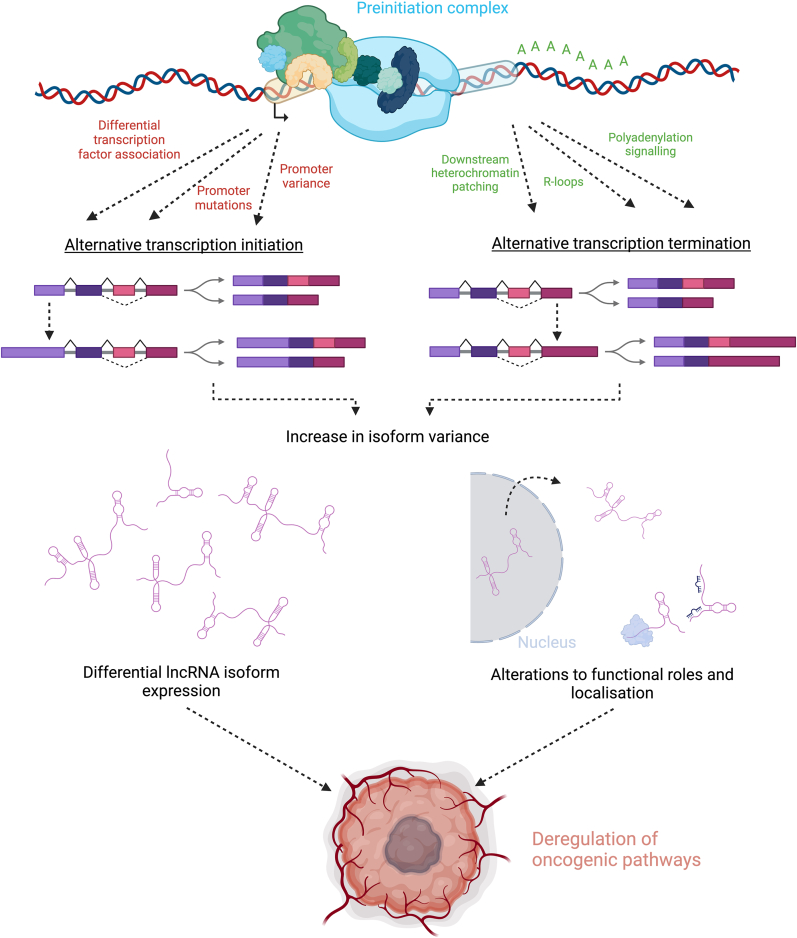
Transcriptional events lead to increased isoform variation, structurally different lncRNA have different oncogenic properties. **A.** Multiple factors can influence transcription initiation and termination resulting in alternative 3′ and 5′ regions, this in conjunction with splicing increases the complexity of lncRNA isoforms. **B**. LncRNA function is dictated by its sequence and structure, as variation occurs the roles of each isoform may differ as is discussed in the body of this article. **C.** In the case of many lncRNA there is oncogenic potential for specific isoforms of the same gene dependent on their differential expression and altered function. (Created in BioRender. Bone, M (2025)).

The Growth Arrest-Specific 5 (*GAS5*) gene encodes one of the most extensively studied lncRNAs in cancer. Initially identified as a downregulated transcript in breast cancer in 2009 [88], GAS5 has since emerged as both a diagnostic biomarker and a promising therapeutic target [89]. Beyond breast cancer, GAS5 is frequently downregulated across a broad spectrum of malignancies, where its loss is associated with dysregulation of cell cycle control, an event that can critically contribute to tumour development and progression [[90], [91], [92], [93]]. GAS5 is transcribed into a multitude of RNA isoforms, with the latest T2T CHM13v2.0/hs1 genome assembly (as annotated by UCSC) listing 31 structural variants and one antisense lncRNA. While many of these isoforms result from alternative splicing events, several also differ due to the use of alternative transcription start sites. Additionally, more subtle variations are observed at the 3′ end, where differences in exon length and position, often by just a few nucleotides arise from alternative transcription termination mechanisms. To date few studies have delved into molecular depth as to how different known GAS5 isoforms may be responsible for inhibiting oncogenic processes. A well-studied phenomenon within this gene is that the alternative splicing of GAS5 at exon 7 produces two main isoforms: GAS5a (short exon 7) and GAS5b (long exon 7). These sequence differences can alter the thermodynamic properties of the RNA, including folding enthalpy and entropy, which together determine secondary structure stability, influencing GAS5's regulatory capacity in cancer-related pathways [[94], [95], [96], [97]]. While direct comparisons of GAS5 isoform thermodynamics are limited, computational studies suggest that even small sequence shifts can significantly impact RNA structural ensembles and function [98]. Although this splice induced isoform variation has been studied extensively, there remains many avenues of exploration into the roles of subtle alternative transcription variation impacting its roles in cancer and normal cellular pathways such as proliferation. The terminal exon 12-derived stem-loop of lncRNA GAS5 mimics the DNA based glucocorticoid response element (GRE), enabling it to bind the glucocorticoid receptor (GR) and block its interaction with DNA. This decoy mechanism suppresses GRE-driven gene expression, such as cIAP2, and promotes apoptosis during cellular stress, pathways highly relevant in the field of cancer research [99,100]. As there are many variants of GAS5 with alternatively recognised lengths of exon 12 [101], further insights into how alternative transcription influences subtle structural changes to this GRE mimic will be essential in uncovering GAS5 functional roles. With the small molecule NPC86 currently employed to target this region of GAS5 in alternative diseases such as neurodegenerative inflammation, understanding which aspects of this molecule and specific isoforms are critical for its function have never been more relevant [102,103] (see Fig. 1).

#### 1.2.2 LINC00941

**Fig. 2:**
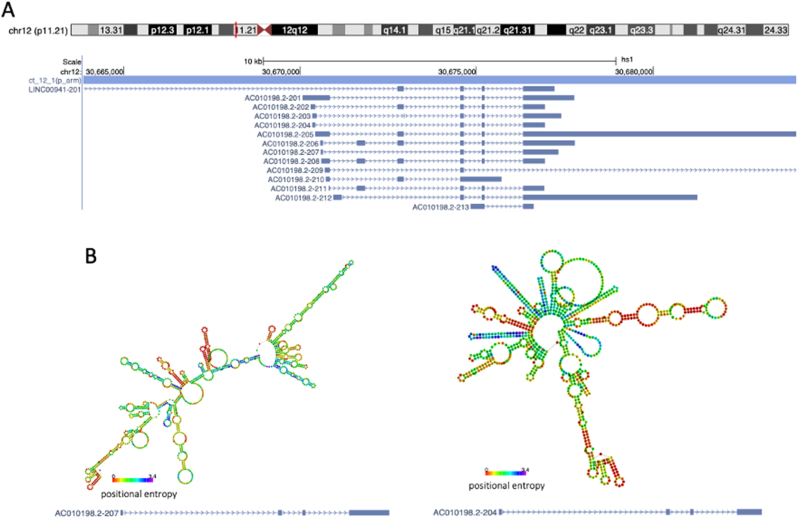
The 3′ and 5′ variation of similarly spliced LINC00941 isoforms alters their secondary structure. **A.** The genome location and identified isoforms of LINC00941 (AC01098.2) obtained from UCSC Genome Browser [185]. **B.** Structural variations of LINC00941 isoforms 204 and 207, generated using minimum free energy models on RNAfold [109].

The lncRNA gene LINC00941 has been implicated as functional in multiple cancers. In pancreatic cancer it promotes progression by sponging miR-335-5p and thus in turn alters ROCK1-mediated LIMK1/Cofilin-1 signalling pathways, increasing proliferation and invasiveness [104]. In normal tissue homeostasis it is associated with moderating epidermal-mesenchymal transitions by repressing the *SPRR5* gene [105]. In terms of nuclear function, it is identified as acting as a chromatin looper, and transcriptional regulator, such as by mediating ILF2 and YBX1 in oesophageal cancer. In the cytoplasm it can stabilize both mRNA and proteins, including the ANXA2 protein in pancreatic cancers to suppress its degradation via NEDD4L [[106], [107], [108]]. Despite multiple roles attributed to this gene, little evidence exists to suggest which isoforms may be playing specific roles and how exonic regions may harbour oncogenic properties. AC01098.2 is synonymous with LINC00941 isoforms transcribed at alternate 5′ start sites with varying degrees of polyadenylation (Fig. 2A). The study of LINC00941'*s* regulation of normal human epidermal homeostasis and much of its cancer promoting activity, such as its interactions with ANXA2, were carried out with relation to the conserved terminal exon sequence across all LINC00941 isoforms [[105], [106], [107], [108]]. While instrumental in elucidating the functionalities of these isoforms, scrutinizing the splicing patterns and alternative transcription mechanisms within these molecules could yield further distinctions between those harbouring physiological functions and those manifesting pathogenic attributes. Within isoforms containing the same exons there can arise different lengths of terminal and proximal exons from ATI and ATT. Using the online software RNA fold which predicts the secondary structure and entropy of sequences in a molecule, we can see that this alternative transcription can create different structural variations dependent on the sequence changes of these isoforms [109] (Fig. 2B). With lncRNA function directly linked to the sequence and structure of a molecule, exploring the splicing and alternative transcription of LINC00941 may be key to distinguishing which molecule are enacting the oncogenic roles of this gene. Many additional factors including protein interactions can further impact folding dynamics exhibited by specific lncRNA, both sequence variation and other folding pathways are poorly characterised thus far in LINC00941 and most other lncRNA and represent a sparsity of knowledge surrounding molecular mechanisms potentially essential for the oncogenic roles [[110], [111], [112], [113], [114]].

### 1.3 Alternative transcription drives the formation of complex secondary structures including the circularization of lncRNA

Transcriptional variance can result not only in sequence alterations but also in substantial changes to the secondary structure of lncRNA molecules. Alternative transcription events, including the use of distinct transcription start or termination sites, can generate isoforms with differing exon compositions or lengths, which in turn affect the folding landscape of the RNA. These structural shifts may alter binding affinities, stability, or subcellular localization. In some cases, such transcriptional variation facilitates the formation of complex secondary structures, including circularised lncRNAs (circRNAs), which are produced through back-splicing mechanisms and often exhibit enhanced stability and distinct functional properties compared to their linear counterparts [[115], [116], [117]].

#### 1.3.1 PVT1

**Fig. 3:**
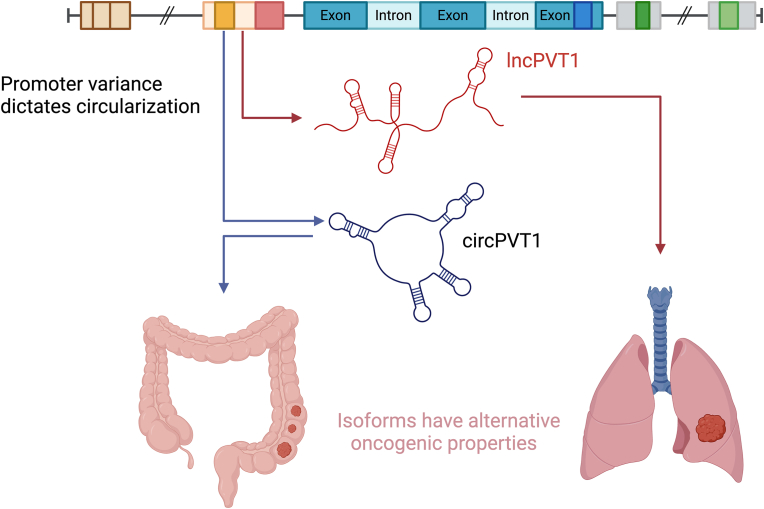
The ATI of PVT1 can determine the circularization of the molecule. LncPVT1 and CircPVT1 arise from splicing, which is determined by transcriptional events, both isoforms enact alternative oncogenic roles as is discussed in the main body of this article. (Created in BioRender. Bone, M (2025)).

This is apparent in one of the most extensively studied lncRNA in the field of cancer, Plasmacytoma Variant Translocation 1 (*PVT1*), a gene which is situated 54 kb downstream of the c-myc proto-oncogene locus of which it is recognised as having a feedback regulatory mechanism with [[118], [119], [120]]. The *PVT1* locus has 190 isoforms attributed to it on the Ensembl database, which include both circular and linear transcripts of varying lengths referred to as lncPVT1 and circPVT1 (Fig. 3) [62,120,121]. These distinct structural isoforms are recognised as having unique functional roles in cancer, circPVT1 has specifically been recognised as a proliferative factor and prognostic marker in gastric cancer predictably by sponging the tumour suppressor miR-125 [122]. The linear counterpart is linked to specific functional roles including the promotion of angiogenesis by sponging miR-29c and altering the vascular endothelial growth factor (VEGF) signalling pathway in non-small-cell lung cancer [121,123]. Within the multitude of PVT1 isoforms, specific regions have been attributed to oncogenic functions, such as exon 2 being identified as having a unique role in cutaneous squamous cell carcinoma oncogenesis [124]. Although much of the variance in differential PVT1 isoforms is generated from splicing, the ATI and ATT play particular roles of importance when considering the formation of circPVT1 and transcripts that include/exclude the oncogenic exon 2 [125]. The formation of circPVT1 is generated by circularization from exon 2 of the *PVT1* gene which then loops via back splicing [126]. This process however is mediated by the use of alternate promoter sequences, regulated exclusively via the mut-p53/YAP/TEAD complex in head and neck squamous cell carcinoma and is therefore a principal product of ATI [[127], [128]]. With circPVT1 having unique oncogenic roles and relative abundances, understanding more completely the factors that lead to alternative oncogenic PVT1 isoforms is paramount in developing targeted therapies for this molecule.

### 1.4 Transcriptional events influence subcellular lncRNA localization

As has been extensively previously explored, lncRNA nuclear retention can be dictated by both 5′ 7-methyl guanosine capping, and 3′ polyadenylation [[129], [130], [131], [132], [133]]. Due to alternative transcription, 5′ and 3’ regions of a gene are lengthened or shortened generating isoforms with unique features that can influence the subcellular distribution of lncRNAs, directing them to various cellular compartments such as the nucleus, cytoplasm, or organelles. The differential localization of lncRNA isoforms is crucial, as it allows them to perform diverse functions in different cellular contexts such as enacting miRNA sponging specifically in the cytoplasm or interacting with chromatin specifically in the nucleus to mediate epigenetic expression of other RNA [[40], [129]].

#### 1.4.1 CCAT1

**Fig. 4:**
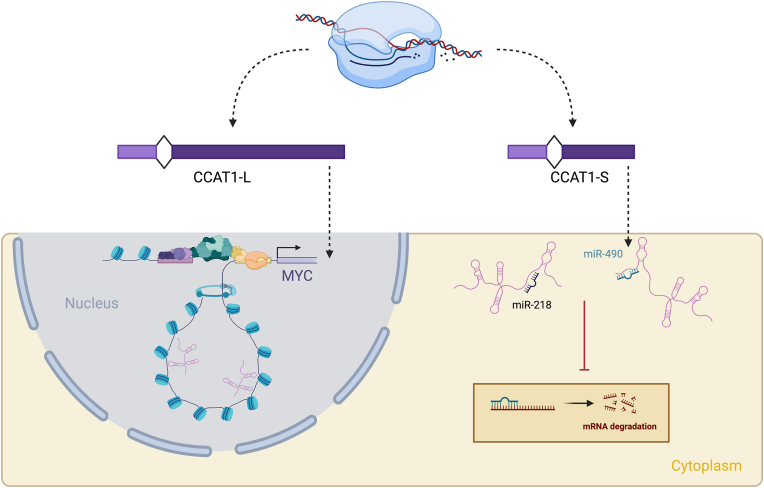
The ATT of CCAT1 dictates the localization and oncogenic roles of the RNA. Top panel: CCAT1-long and short (L and S) isoforms arise from alternative transcription termination events. Bottom panel: CCAT1-L acts as an oncogenic super-enhancer of MYC via chromatin looping, CCAT1-S is an oncogenic driver as it is a miRNA sponge, preventing miRNA degradation of mRNAs encoding tumour suppressor proteins. (Created in BioRender. Bone, M (2025)).

The colon cancer associated transcript 1 (*CCAT1*) is a lncRNA gene that exists in the sparsely protein coding and highly cancer associated locus of 8q24.21, where both *MYC* and *PVT1* also reside [62]. CCAT1 is formed as an antisense lncRNA and consists of two identified isoforms with 2 exonic regions, the 2600 nt CCAT1-S and the 5200 nt CCAT1-L which are formed by the extension of the exon at its 3′ end [134]. These isoforms were initially identified as being highly expressed in multiple cancers across many patients, including colorectal, and having specific localisations, with CCAT1-L being retained in the nucleus and CCAT1-S localising to the cytoplasm (Fig. 4) [[134], [135], [136], [137], [138]]. Nuclear-retained CCAT1-L functions not only as a cancer biomarker but also as a key driver of tumorigenesis, primarily through its regulation of the MYC oncogene. It directly interacts with the RNA-binding protein CTCF, which is enriched at chromatin loop formation sites. Through this interaction, CCAT1-L facilitates chromatin looping between the *MYC* promoter and its upstream enhancers, effectively acting as a super-enhancer to promote MYC expression [134,[139], [140], [141]]. Downstream effects of the upregulation of CCAT1-L in cancer can include promoting the invasiveness of ovarian cancer, worsening disease prognosis [142]. In the cytoplasm CCAT1-S is also highly linked to several oncogenic pathways, it can act for a microRNA sponge as is the case in non-small cell lung cancer where has been identified as a competing endogenous RNA to miR-218, miR-490, and miR-216a-5p [[143], [144], [145]]. Although the mechanisms for nuclear or cytoplasmic retention for both CCAT1 isoforms have yet to be fully understood, it is apparent that the alternative 3′ ends of the molecule directly dictate its localization. As a pivotal mechanism in the localization of this oncogenic lncRNA, alternative transcription unveils the potential for numerous undiscovered roles in the localization of the estimated 300,000 lncRNA transcripts. These roles extend to both normal tissue homeostasis and cancer, suggesting a broader landscape of functional implications.

### 1.5 Current methods for distinct lncRNA isoform quantification

As highlighted in this review, the diverse isoforms arising from alternative transcription start and end events significantly complicate the targeted study of lncRNAs in both in vitro and in vivo settings, as well as their potential therapeutic applications in cancer. A lack of definition in specific 3′ and 5′ ends can result in incomplete or ambiguous transcript annotations, leading to challenges in accurately detecting full-length lncRNA isoforms. Standard short-read sequencing may miss crucial transcription start or termination sites, especially in cases where lncRNAs lack well-defined polyadenylation signals. This can lead to fragmented transcript reconstruction, misassignment of exons, or even the exclusion of certain isoforms from reference databases [[146], [147], [148], [149]]. Several technologies however exist to allow for RNA quantification at an isoform specific level, which holds high relevance for lncRNA isoform detection arising from both transcriptional alterations and splicing.

#### 1.5.1 CAGE_seq

Cap Analysis of Gene Expression sequencing (CAGE-seq), first introduced by Shiraki et al., in 2003, is a high-throughput sequencing technique designed to map TSSs with high precision. The method involves isolating and sequencing concatemers of DNA tags derived from the first ∼20 nucleotides of the 5′ ends of capped RNAs, ensuring that only actively transcribed, capped mRNAs and lncRNAs are captured. By providing a quantitative measure of TSS usage across the genome, CAGE-seq enables accurate promoter identification, distinguishes between alternative TSSs, and reveals tissue- or condition-specific promoter activity. This makes it particularly useful for studying lncRNA regulation, enhancer activity, and dynamic transcriptional changes in diseases such as cancer [[150], [151], [152]]. CAGE-seq does however have its limitations, principally in its specificity in detecting processed and capped RNA molecules, the cap specific enzymes typically used in CAGE (such as Tobacco Acid Pyrophosphatase, TAP) would be incapable of recognising uncapped lncRNA making it an inappropriate means of detection for a large proportion of these molecules.

#### 1.5.2 Long read sequencing

Long read (LR) sequencing is another RNA sequencing technology advancing in recent years that offers several advantages to standard sequencing in terms of isoform coverage and offer a more extensive alternative transcription read potential than older CAGE-seq methodologies [153]. Most notably highlighted by studies from the Long-read RNA-Seq Genome Annotation Assessment Project Consortium, LR-seq has the capacity to predict ATS and ATT variances with greater accuracy than previously possible and in a wealth of human tissues [154]. Currently available long-read sequencing (LR-seq) technologies, such as PacBio SMRT sequencing and Oxford Nanopore Technology, offer the capability to sequence full-length transcripts with high accuracy. PacBio SMRT (Single Molecule, Real-Time) sequencing utilizes a circular consensus sequencing approach, where the same molecule is read multiple times to improve base-calling accuracy. This enables precise detection of alternative isoforms, complex splicing events, and full-length lncRNAs, reducing errors associated with transcript reconstruction in short-read sequencing. Alternatively, Oxford Nanopore (ONT) sequencing can directly sequence native RNA or cDNA by passing molecules through a biological nanopore, detecting nucleotide sequences based on characteristic electrical signal changes. Unlike short-read methods, ONT can generate reads exceeding 100 kb, making it particularly advantageous for capturing long and highly structured lncRNAs, distinguishing closely related isoforms, and detecting post-transcriptional modifications such as m6A methylation. By eliminating the need for transcript assembly and enabling the detection of complete lncRNA structures, alternative transcription start/stop sites, and overlapping isoforms, these LR-seq technologies significantly enhance the accuracy of lncRNA annotation, isoform quantification, and functional analysis in cancer and other diseases as has already been done with the detection of aberrant mRNA transcripts [155].

### 1.6 Investigative approaches and therapeutic considerations for alternative lncRNA isoforms

The modulation of lncRNA expression is essential from both the context of understanding the underlying functional mechanisms regarding a lncRNA and paving the way for potential therapeutic treatment avenues of oncogenic molecules. There are two principal strategies in oncological lncRNA therapeutics: silencing oncogenic lncRNAs that are overexpressed in cancer and restoring the expression of tumour-suppressive lncRNAs that become downregulated as the disease progresses.

#### 1.6.1 Removal of lncRNA

**Fig. 5:**
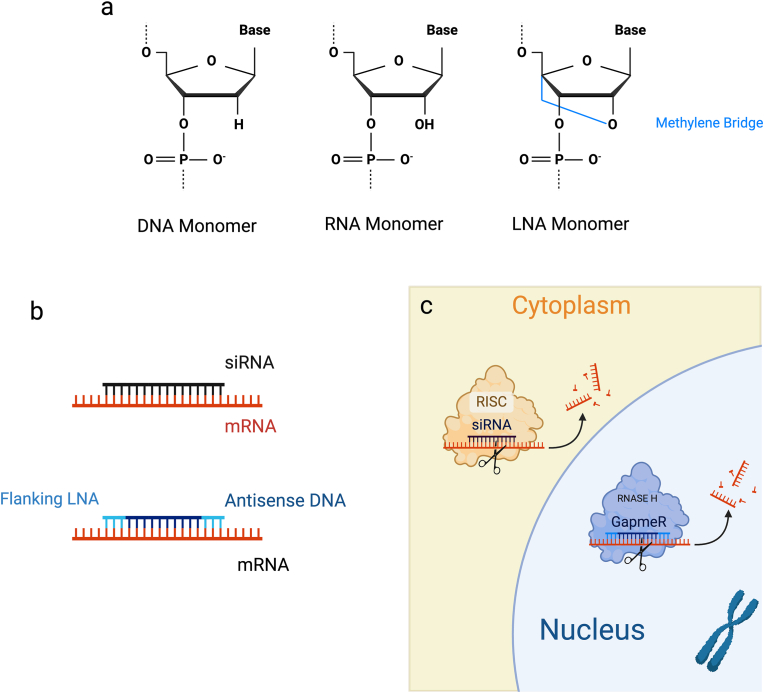
Antisense oligonucleotide (ASO) GapmeR schematic and utility. A) The standard structure of a DNA, RNA, and locked nucleic acid (LNA) used for flanking regions of a GapmeR molecule. B) The approximate composition of any designable GapmeR molecule compared to an siRNA. C) The nuclear activity of GapmeRs and the recognised degradation pathways of mRNA bound to both siRNA and GapmeRs by the RISC complex and RNaseH respectively. (Created in BioRender. Bone, M (2025)).

Antisense oligonucleotides (ASOs) stand out as a promising therapeutic avenue in cancer treatment, specifically regarding the intricate world of silencing pathological mRNA expression. Predominantly they are short single stranded oligodeoxynucleotides, synthetically generated to match a specific RNA sequence and bind with increased affinity due to structural modifications such as 2′O – methyl and 2′O – methoxyethyl additions [[156], [157], [158]]. These molecules can target both cytoplasmic and nuclear RNAs via RNaseH mediated degradation making them of therapeutic interest in the targeting of various lncRNA [[159], [160], [161]]. The highly targeted nature of ASOs not only maximizes their therapeutic efficacy but also minimizes the potential for off-target effects, thereby reducing the risk of adverse reactions. Currently several ASOs have been FDA approved as treatments for various diseases such as nusinersen which disrupts the protein coding SMN1 RNA to mitigate the effects of spinal muscular atrophy [162]. To date there are no commercially available ASO for the treatment of cancer, or which specifically target lncRNA however in both instances there are molecules in early stage clinical trials [61]. Among these, Locked Nucleic Acid (LNA) GapmeRs have emerged as a particularly promising approach. LNA modifications enhance the stability and target affinity of ASOs by introducing a conformationally restricted ribose structure, increasing resistance to nuclease degradation. This makes LNA GapmeRs highly effective in targeting oncogenic lncRNAs, leading to their degradation via RNaseH-mediated cleavage (Fig. 5) [[163], [164], [165]]. As our understanding of the intricate regulatory networks governed by lncRNAs deepens, the development and optimization of ASOs targeting specific lncRNAs hold great potential for personalized cancer therapies. As this research advances, isoform variance will hold particular importance, as has been discussed earlier lncRNA are capable of containing both essential and pathogenic isoforms such as in the case of *MALAT1*. In the field of drug design, a specific focus should be directed towards the isoform variance of lncRNA, where alternative transcription and splicing emerge as pivotal factors dictating events in cancer. ASOs are not solely limited to their silencing function in lncRNA research; they also serve as powerful tools for functional and mechanistic studies. Beyond transcript knockdown, ASOs can be employed in pull-down assays to investigate lncRNA-protein, lncRNA-RNA interactions, and the quantity of specific lncRNA transcripts. This approach is particularly valuable when prior silencing experiments reveal tumour-suppressive effects, suggesting a functional role for the targeted lncRNA in cancer progression. An elegant use of this approach, performed by Montes et al., 2021, leveraged streptavidin coupled ASO's to pull down MIR31HG and detect interacting molecules involved with senescence pathways [166]. This technology could theoretically be coupled with adjacent LR-seq to ensure specific lncRNA isoforms being investigated are indeed those responsible for oncogenic roles observed.

In addition to the methods discussed for modulating lncRNA expression, it is worth highlighting CRISPR-Cas13 as a promising tool for isoform-specific targeting. Unlike Cas9-based systems that modify genomic DNA, Cas13 enzymes target RNA directly, enabling precise post-transcriptional regulation without introducing permanent changes to the genome. This feature is particularly advantageous in therapeutic contexts, as it eliminates the risk of germline transmission and off-target genomic edits. Given its capacity to degrade or modulate specific RNA molecules in a transient and reversible manner, CRISPR-Cas13 offers a valuable platform for investigating the function of distinct lncRNA isoforms and may serve as a complementary approach to antisense oligonucleotide technologies in the development of RNA-targeted cancer therapeutics [[167], [168], [169], [170]]. Overall, targeting oncogenic lncRNAs for removal remains one of the most promising and widely explored strategies for future therapeutic intervention. However, a detailed understanding of how alternative transcription influences the structure and function of specific lncRNA isoforms will be critical in determining which variants should be selectively targeted.

#### 1.6.2 Overexpressing lncRNA

In contrast to the silencing of oncogenic lncRNAs, the restoration of tumour-suppressive lncRNAs presents an even greater challenge due to the complexity of isoform diversity and the structural sensitivity of lncRNAs to minor sequence variations. Unlike protein-coding genes, where restoring function often involves reintroducing a specific sequence, lncRNA function is dependent on their secondary and tertiary structures which impact RNA-protein interactions, and subcellular localization. As outlined previously slight sequence alterations can lead to significant structural and functional changes. The lack of current comprehensive isoform characterization makes it difficult to determine which specific variant need to be restored for therapeutic efficacy, technologies such as LR-seq may improve the accuracy of correctly identifying the distinct RNA code required to restore a tumour suppressive lncRNA molecule. Achieving feasible overexpression of lncRNAs presents several technical challenges that must be addressed. While lentiviral-mediated overexpression is commonly used to upregulate both lncRNAs and mRNAs in experimental studies, this approach inherently modifies the nascent RNA sequence, potentially altering its structure and function [[171], [172], [173]]. Most apparent of such modifications to the nascent RNA are the addition of lentiviral transfection included synthetic or viral-derived 3′ untranslated regions (UTRs) for transcription termination which although don't offer structural differences for proteins encoded by genes overexpressed in this manner, will affect lncRNA sequences [[174], [175], [176]]. Another commonly used overexpression tool with limitations in lncRNA biology is CRISPR activation (CRISPRa). CRISPRa works by using a catalytically inactive Cas9 (dCas9) fused to transcriptional activators, such as VP64, p65, or VPR, to upregulate gene expression without cutting DNA. A guide RNA (gRNA) directs dCas9 to the promoter or enhancer region of the target lncRNA, recruiting transcriptional activators to enhance its endogenous expression [[177], [178], [179], [180]]. This method preserves natural splicing, isoform diversity, and regulatory elements, making it a more physiologically relevant approach for lncRNA overexpression compared to lentiviral transfections, which often introduce exogenous promoters and artificial 3′ UTRs, modifying native lncRNA sequences [181].

However, CRISPRa cannot selectively overexpress distinct lncRNA isoforms, as it activates transcription at the gene locus rather than controlling alternative splicing or polyadenylation site selection. This means that all endogenously transcribed isoforms may be upregulated simultaneously, making it unsuitable for studies requiring the exclusive overexpression of a specific transcript variant. An emerging and promising alternative to lentiviral and CRISPR-based methods for lncRNA overexpression in cancer research is the use of transposon-based systems, such as Expression of LncRNAs with Endogenous Characteristics using the Transposon System (ELECTS) [182]. This method primarily involves integrating lncRNA sequences into the genome via transposable elements, allowing for stable, long-term expression while preserving native regulatory elements, isoform diversity, and physiological transcriptional control. Vectors are typically co-transfected with transposons that contain complementary elements, such as piggyBac, and are equipped with inducible promoters-such as doxycycline-inducible systems-to enable precise, controlled expression of a specific, structurally accurate lncRNA isoform [176,183,184]. This approach allows for the overexpression of a specific and structurally accurate lncRNA isoform in a permanent yet inducible manner. By avoiding viral promoters and artificial UTRs, ELECTS provides a more accurate representation of endogenous lncRNA function, making it a valuable tool for studying alternative lncRNA isoforms in cancer.

## 2 Conclusion

Long non-coding RNAs (lncRNA) remain a highly promising domain of cancer research, serving as both biomarkers and key contributors to the development of various neoplastic diseases. As ASO therapeutics continue to advance, it is increasingly anticipated that clinical trials targeting lncRNAs will become more prevalent, particularly as adjuncts to established cancer treatments such as chemotherapy, radiotherapy, and surgical intervention. This combined approach may enhance therapeutic efficacy by selectively silencing oncogenic lncRNA isoforms that contribute to treatment resistance and disease progression. Crucial to the future of lncRNA research and the development of potential therapeutic interventions is a profound understanding of the significance of isoform variation. This variation plays a pivotal role in determining whether an RNA molecule acts as a pathogenic factor or an essential homeostatic regulator. By delving into the intricacies of splicing and alternative transcription mechanisms that govern the structural and functional activities of lncRNAs, we can more accurately attribute functional roles to specific isoforms of a given gene. This approach will not only enhance our comprehension of lncRNA functionality but also may pave the way for a more precise targeting strategy. Rather than focusing on specific genes, the emphasis shifts toward isoform specificity. This precision promises to advance research in this complex and elusive field, bringing us closer to breakthroughs in the treatment of cancer through targeted lncRNA interventions.

## CRediT authorship contribution statement

**Max Bone:** Writing – original draft, Investigation, Conceptualization. **Gareth J. Inman:** Writing – review & editing, Supervision.

## Funding

M.B. was supported by a British Skin Foundation PhD studentship (004_S_19) and Cancer Research UK core funding to the CRUK Scotland Institute (A31287). G.J.I. and members of his laboratory were supported by Cancer Research UK core funding to the CRUK Scotland Institute and Cancer Research UK core programme funding to G.J.I. (A29802).

## Declaration of competing interest

The authors declare that they have no known competing financial interests or personal relationships that could have appeared to influence the work reported in this paper.
